# Participation in Physical Education Classes and Health-Related Behaviours among Adolescents from 67 Countries

**DOI:** 10.3390/ijerph19020955

**Published:** 2022-01-15

**Authors:** João Martins, Adilson Marques, Élvio Rúbio Gouveia, Francisco Carvalho, Hugo Sarmento, Miguel González Valeiro

**Affiliations:** 1 Centro de Estudos de Educação, Faculdade de Motricidade Humana e UIDEF, Instituto de Educação, Universidade de Lisboa, 1649-004 Lisboa, Portugal; franciscocarvalho8@hotmail.com; 2 Facultad de Ciencias del Deporte y la Educación Física, Universidad de A Coruña, 15001 A Coruña, Spain; maglez@udc.es; 3 CIPER, Faculty of Human Kinetics, University of Lisbon, 1649-004 Lisboa, Portugal; amarques@fmh.ulisboa.pt; 4 ISAMB, Faculty of Medicine, University of Lisbon, 1649-004 Lisboa, Portugal; 5 Departement of Physical Education and Sport, University of Madeira, 9000-072 Funchal, Portugal; erubiog@staff.uma.pt; 6 Interactive Technologies Institute, LARSyS, 9020-105 Funchal, Portugal; 7 Research Unit for Sport and Physical Activity (CIDAF), Faculty of Sport Sciences and Physical Education, Estádio Universitário de Coimbra, University of Coimbra, 3040-256 Coimbra, Portugal; hugo.sarmento@uc.pt

**Keywords:** physical education, physical activity, sedentary behaviour, nutrition, smoke, alcohol, healthy lifestyle, adolescence, preventative health, public health

## Abstract

The present study sought to examine the associations between participation in physical education (PE) classes and a range of health-related behaviours among adolescents. Secondary analysis of self-reported data from the Global Student Health Survey, collected between 2010 and 2017 from 222,121 adolescents (N = 117,914 girls; 49.0%; aged 13–17 years) from 67 countries and five world regions, was carried out. Participation in PE classes (0, 1–2, ≥3 days/week) was the independent variable. Physical activity (PA); sedentary behaviour (SB); active travel to school; fruit, vegetables, and alcohol consumption; and smoking; as well as adopting ≥5 of these healthy behaviours; were the dependent variables. Complex samples logistic regressions were performed to explore the associations between participation in PE classes and health-related behaviours. The results revealed that 18.2% of adolescents did not take part in PE classes. A total of 56.7% and 25.1% of adolescents reported participating in PE classes on 1–2 and ≥3 days/week, respectively. Only 26.8% of adolescents adopted ≥5 healthy behaviours. Participation in PE classes was positively associated with PA, active travel, fruit consumption, and vegetable consumption (only for ≥3 days/week), but was negatively associated with meeting SB recommendations, and with not smoking (only for girls and ≥3 days/week). Overall, PE participation was positively associated with adopting ≥5 healthy behaviours, with favourable results found for those who attended more PE classes. The findings revealed a positive association between participation in PE classes and a range of health-related behaviours among adolescents. This suggests that, worldwide, quality PE should be delivered at least 3 days per week up to daily to promote healthy lifestyles among adolescents.

## 1. Introduction

Worldwide, many adolescents adopt unhealthy lifestyle behaviours [1,2,3,4] and fail to meet recommendations regarding physical activity (PA) [5,6], sedentary behaviour (SB) [7,8,9,10], nutrition, smoking, and alcohol consumption [2,3,11,12,13]. Furthermore, adopting unhealthy behaviours during childhood and adolescence can track into adulthood [14], presenting risks for current health and the development of chronic diseases later in life [15,16]. Thus, from a public health and educational point of view, promoting healthy lifestyle behaviours among young people is important for improving health and preventing chronic diseases.

In this regard, the important role that schools, and particularly physical education (PE), can play in students’ healthy development has become increasingly acknowledged in research [17,18,19,20] and policy [6,21,22,23,24,25]. In fact, triggered by concerns over adolescents’ health, providing students with the knowledge, skills, and attitudes to adopt and maintain physically healthy and active lifestyles throughout life has become a key and widely agreed aim of PE [20,22,23].

Current evidence suggests that PE provides many benefits and positive outcomes for students, such as higher PA levels, physical fitness, fundamental movement skills, and improved cognition [22,26,27,28,29,30]. One important component that has been explored in the literature is the time allocated to curriculum PE and the number of weekly PE classes [23,28,29,31,32]. In fact, higher levels of participation in PE classes have been consistently associated with the adoption of more favourable physical activity patterns [22,33,34,35]. Furthermore, participation in PE classes has been associated with lower levels of SB in some studies [36,37,38] but not in others [39,40]. Thus, further research to explore the relationships between PE and SB and the moderating roles of age, sex, socioeconomic status, and country income have been recommended [39,40,41]. Similarly, limited evidence is available regarding the link between PE and nutrition, smoking, and alcohol consumption [22,34].

Although evidence supports schools and PE as key contexts for learning about and making healthy choices [6,20,22,23,24,25], little is known about the association between participation in PE classes and the adoption of healthy lifestyles. This is particularly true when considering nationally representative samples of adolescents from low- and middle-income countries from different world regions. Previous studies also focused mainly on the relationship between PE and one or two health-related behaviours, and notably PA [33,35]. Considering that unhealthy behaviours co-occur in adolescence [2,3,4], an improved understanding of multiple health-related behaviours could inform the development and implementation of more effective interventions and strategies for promoting healthier lifestyles in school and PE contexts. Thus, the present study aimed to investigate the relationship between participation in PE classes and meeting different health-related behaviour recommendations, and with a healthy lifestyle index, considering important factors, such as gender, age, socio-economic status, country income, and world regions, in nationally representative samples of adolescents from 67 countries.

## 2. Methods

### 2.1. Data Source and Participants

Secondary data collected from the Global School-Based Student Health Survey (GSHS) (http://www.who.int/chp/gshs/datasets/en/; accessed on 17 September 2020) between 2010 and 2017, were used in the present study. This standardized school-based and cross-sectional survey aims to assess the behavioural risk factors and protective factors among young people aged 13–17 years from several countries worldwide. The GSHS was developed by the World Health Organization (WHO) and the Centers for Disease Control and Prevention (CDC) in collaboration with the United Nations Children’s Fund; the United Nations Educational, Scientific, and Cultural Organization (UNESCO); and the Joint United Nations Programme on HIV/AIDS. In each country, the GSHS used a standard school-based methodology and a standardized two-stage sample selection to collect representative data of adolescent students, as well as core questionnaire modules. To ensure that the surveys are standardized, and thus comparable across countries, the highest quality of ongoing data capacity building is provided by WHO and CDC. These processes involve helping with sample design and selection; training of survey coordinators for workshops on survey implementation, data analysis, and reporting; provision of implementation handbooks and other materials; and data entering, editing, and weighting processes. Data collection was, therefore, coordinated and carried out by previously trained staff. Further details on the GSHS methodology can be found elsewhere [42].

From the available data, all nationally representative data sets with more than 99 participants that included the following variables were selected: PE participation, gender, age, “weight”, “stratum”, and “primary sampling unit”. As the survey was primarily designed for adolescents aged 13–17 years following previous research [31], students aged 11, 12, and 18 years were excluded from the analysis. The final sample included 222,121 adolescents (N = 117,914 girls; 49.0%; aged 13–17 years) from 67 countries and five world regions (Africa, Americas, Southeast Asia, Eastern Mediterranean, Western Pacific). Based on the World Bank Classification (data 2010–2017; https://www.worldbank.org; accessed on 3 October 2020), 11 countries had low-income economies, 25 had lower-middle economies, 18 had upper-middle economies, and 10 had high-income economies. Anguilla, Cook Islands, and Niue were not classified in this regard (cf. Supplementary Table S1).

### 2.2. Instrument and Measures

The GSHS questionnaire is comprised of 10 core questionnaire modules addressing the leading causes of morbidity and mortality worldwide. The GSHS questionnaire was completed during one regular class period, voluntarily and privately. Only students who had previously provided written or verbal consent, and written consent from their parents participated in the study. All procedures were performed in accordance with the ethical standards of the 1964 Helsinki declaration and its later amendments or comparable ethical standards.

For measuring participation in PE classes, students were asked: “During this school year, on how many days did you go to physical education class each week?” The response options were: 0 days, 1 day, 2 days, 3 days, 4 days, and 5 or more days. As in previous studies [35,39,43], PE participation was recoded into three categories: 0 days/week, 1–2 days/week, and ≥3 days/week.

PA was assessed using a valid and reliable question [44]: “During the past 7 days, on how many days were you physically active for a total of at least 60 min per day?” Answers were given on an 8-point scale, ranging from 0 to 7 days. Based on the WHO guidelines [6], students were classified as “sufficiently active” if they did at least 60 min of PA on all seven days of the week, as in previous research [2,35,43].

Sedentary behaviour (sitting time) was measured by asking: “When you are not at school or doing homework, how much time do you spend during a typical or usual day sitting and watching television, playing computer games, talking with friends, or doing other sitting activities?” Based on the 24 hours movement guidelines [9], low SB (sitting time) was defined as ≤2 h/day [7].

Active travel to school was assessed by asking the students: “During the past 7 days, on how many days did you walk or ride a bicycle to or from school?” Response options ranged from 0 to 7 days. Those students who reported actively travelling to school on at least 1 day were considered to be active travellers [45].

Fruit consumption was measured with the question: “During the past 30 days, how many times per day did you usually eat fruits?” Meeting fruit consumption recommendations was defined as having fruit ≥2 times per day during the past 30 days [2,12].

Vegetable consumption was assessed with the question: “During the past 30 days, how many times per day did you usually eat vegetables?” Meeting vegetable consumption recommendations was defined as having vegetables ≥3 times per day during the past 30 days [2,12].

For cigarette smoking, the question posed to the students was: “During the past 30 days, on how many days did you smoke cigarettes?” Cigarette smoking was defined as smoking on one or more days in the past 30 days [2,13,46].

Regarding alcohol consumption, the following question was asked: “During the past 30 days, on how many days did you have at least one drink containing alcohol?” Alcohol consumption was defined as having at least one drink containing alcohol in the past 30 days [2,13].

A composite score for a healthy lifestyle index was then created by taking all questions into account. Students earned a point if they met each of the following criteria for each health-related behaviour: (a) 60 minutes of daily PA, (b) spending less than two hours a day in SB (sitting time) during leisure time, (c) active travel to school once or more per week, (d) eating fruit 2 or more times/day, (e) eating vegetables 3 or more times/day, (f) no alcohol consumption, and (g) no smoking. Thus, the healthy lifestyle index score ranged from 0 to 7 points. Based on criteria adopted in previous research, for analysis purposes, those participants who had 5 or more points were classified as having a healthy lifestyle [2,3].

Regarding sociodemographic characteristics, the students reported their sex (male or female) and age (years). Since the GSHS did not include any specific measures of socioeconomic status, food insecurity (hunger status) was used as a proxy measure like in previous studies [2,35]. In this regard, participants were asked: “During the past 30 days, how often did you go hungry because there was not enough food in your home?” Responses were categorized as follows: “Never/rarely” and “Sometimes/most of the time/always” [10].

### 2.3. Data Analysis

Descriptive data for all participants and variables are presented as weighted percentages and respective 95% confidence intervals (CI). The weighted prevalence estimates of each healthy behaviour and PE classes are also presented by gender. Given the binary nature of each healthy behaviour, logistic regressions were performed to explore the associations between meeting each healthy behaviour and participation in PE classes. These analyses were stratified by gender and adjusted by age, socioeconomic status (hunger status), world region, and country income. Logistic regressions for exploring the relationship between a healthy lifestyle (adopting ≥5 healthy behaviours) and participation in PE classes, stratified by regions and by country income, were also conducted. These analyses were adjusted for each participant’s age and socioeconomic status (hunger status), and conducted separately for boys and girls. In all these analyses, the adjusted estimates of the association parameters are presented in the form of an odds ratio (OR) and 95% CI. In the tables, all cases of OR (95% CI) that are significant are highlighted in bold. All statistical analyses were performed in May of 2021 using the complex samples menu of the IBM SPSS Statistics version 26.0 (IBM, New York, NY, USA) accounting for the “weight,” “stratum,” and “primary sampling unit” (PSU) variables, as recommended by the GSHS protocol [47] to appropriately represent the weighting process and the two-stage sample design. Specifically, the weighting accounted for the distribution of the population by sex and grade, and for the probability of the selection of schools and classrooms, non-responding schools, and students [2,31,47].

## 3. Results

The adolescents’ characteristics are presented in Table 1. A total of 222.121 adolescents aged 13–17 years old (49.0% girls) from 67 countries and five world regions were included in the study. Most adolescents came from a lower-middle-income economic country (60.7%). A total of 56.7% of adolescents participated in PE classes on 1–2 days/week and 25.1% on ≥3 days/week. Most adolescents did not meet current PA (84.8%), vegetable (76.0%), and fruit consumption (63.5%) recommendations. Less than half of the adolescents did not actively commute from home to school (41.0%), 14.6% consumed alcohol, and 9.5% smoked cigarettes in the last 30 days. Only 26.8% of adolescents adopted ≥5 healthy behaviours.

Table 2 presents the prevalence estimates of each healthy behaviour by participation in PE classes for boys and girls. The prevalence of adolescents meeting the PA recommendations increased with the number of PE classes attended by boys (from 11.3% on 0 days to 26.2% on ≥3 days/week) and girls (from 7.8% on 0 days to 16.5% on ≥3 days/week). Boys and girls who participated in PE classes also presented higher prevalence estimates of adopting active travelling modes to school when compared with those who did not participate in PE. A higher prevalence for meeting the SB recommendations was found for those boys (74.8%) and girls (74.7%) who did not participate in PE classes. Boys and girls who reported participating in PE on ≥3 days/week presented a higher prevalence of meeting the vegetable recommendations. For fruit, girls participating in PE classes presented higher prevalence estimates. For smoking and alcohol consumption, similar trends across the different categories of PE participation were found regardless of gender.

Table 3 shows that, compared with adolescents who did not participate in PE, those boys and girls who took PE classes 1-2 days/week (boys: OR 1.76, 95% CI 1.47–2.12; girls: OR 1.67, 95% CI 1.41–1.97) and ≥ 3 days/week (boys: OR 2.75, 95% CI 3.32–3.26; girls: OR 1.67, 95% CI 2.01–2.72) were more likely to meet PA recommendations. Boys and girls who took PE classes also had higher odds of adopting active travel modes to school. However, those boys and girls who did take part in PE classes were less likely to meet SB recommendations (e.g., boys: OR 0.74, 95% CI 0.65–0.84 for ≥3 days/week; girls: OR 0.76, 95% CI 0.68–0.85). Boys and girls who participated in PE classes had somewhat higher odds of meeting the recommendations for fruit consumption. Regarding vegetable consumption, only those adolescents who took PE classes ≥3 days/week (boys: OR 1.36, 95% CI 1.21–1.54; girls: OR 1.42, 95% CI 1.26–1.60) had a higher likelihood of meeting the recommendations. No significant associations were found between participation in PE classes, not smoking, and not drinking alcohol for boys. Girls who took PE classes 1–2 days/week were less likely to smoke (OR 1.26, 95% CI 1.09–1.47). However, an inverse trend was found between girls who participated in PE classes on ≥3 days/week and not smoking (OR 0.71, 95% CI 0.57–0.88) and not drinking alcohol (OR 0.84, 95% CI 0.73–0.97).

Table 4 shows the relationship between the adoption of ≥5 healthy behaviours and participation in PE classes, stratified by world regions. Considering all regions, students who took part in PE classes 1–2 days/week (boys: OR 1.43, 95% CI 1.24–1.64; girls: OR 1.45, 95% CI 1.29–1.63) and ≥3 days/week (boys: OR 2.09, 95% CI 1.77–2.47; girls: OR 1.79, 95% CI 1.58–2.03) had higher odds of adopting ≥5 healthy behaviours. This positive and significant relationship was consistent across each world region for those boys and girls taking PE classes ≥3 days/week, and for most world regions when taking PE classes 1–2 days/week.

Table 5 shows the relationship between adopting ≥5 healthy behaviours and PE participation by country income. When considering all incomes, boys and girls who participated in PE classes were more likely to adopt ≥5 healthy behaviours. This trend applied to the low-, lower-middle-, and upper-middle-income economies/countries. However, for high-income countries, this trend was not identified for boys attending PE classes and for girls attending PE classes 1–2 days/week (OR 1.18, 95% CI 0.85–1.63).

## 4. Discussion

The present study examined the associations between participation in PE classes, health-related behaviours, and a healthy lifestyle using data from national representative samples of adolescent students from 67 countries. Overall, participation in PE classes was positively associated with a range of health-related behaviours. More positive results were found for those who attended more PE classes, regardless of gender, age, socioeconomic status (hunger status), and world region. One exception was the inverse association found between participating in PE classes and meeting SB recommendations. This study also suggested that only one in four adolescents adopted five or more healthy behaviours. In summary, the main results suggested that, worldwide, there is a need to increase the adoption of health-related behaviours among adolescents and, to do so, the implementation of quality PE lessons on a minimum of 3 days/week up to daily might be an important strategy.

The importance of daily PE was highlighted in various policy documents [21,23,32]. In this study, 18.2% of adolescents reported not taking part in any PE classes, whereas 25.1% reported having PE on ≥3 days/week. Considering the benefits of PE [26,28,29,30], it seems critical that all adolescents are provided with quality PE across all school years, and that an increasing proportion of adolescents have daily PE. It is encouraging that this is already happening in some countries (e.g., Hungary) or states/zones within countries [22,31,32].

Consistent with existing evidence [5,48], most adolescents (84.8%) were not sufficiently active and failed to meet the current PA recommendations to benefit their health. Given the current evidence that PE is positively associated with higher levels of PA [22,33,35,40], and that in the present study the likelihood of meeting the PA recommendations increased with the number of PE classes attended for both boys and girls, increasing the time and the number weekly PE classes could be an important strategy to help more adolescents to become more active and healthier. In each lesson, it is important for PE teachers to improve their teaching behaviour, use evidence-based strategies, and provide students with a minimum of 50% of class time in MVPA [18,23,49,50] so that students are provided with a range of quality PE experiences that promote affective, cognitive, social and psychomotor learning [26,28,29,30]. Improved quantity and quality of PE, inserted in a comprehensive school PA approach strategy, has been considered to be critical for helping adolescents to develop their physical literacy and to be better prepared for adopting active and healthy lifestyles for life [23,51,52,53,54].

In the context of a comprehensive approach, one possibility for further increasing PA levels outside of PE classes is by promoting active travel, such as walking and cycling to and from school [24,55]. Active travel can contribute to overall PA and adolescents’ health outcomes [43,55,56,57]. In one of the few studies that, to our knowledge, has specifically explored the association between participation in PE classes and active travel in adolescence, a positive relationship was found [34]. Although the association (and/or mechanisms behind this) cannot be discerned, the results reinforce the importance of providing adolescents with regular weekly PE lessons. PE teachers can use PE time to increase their students’ knowledge about a range of opportunities and contexts within which to be active during the day, help adolescents to identify and overcome specific-contextual barriers to PA participation, and lead initiatives in collaboration with school, community, and family members to promote active travel. Specifically, in PE classes, it might be important to teach students how to ride a bicycle and to stimulate the use of a bicycle as a form of active travel, and of doing PA in leisure time. This is important because the prevalence of this behaviour is low in adolescents from low- and middle-income countries [45]. We acknowledge that innovative teaching approaches and materials are required to introduce this type of content in PE classes. However, this could be an opportunity to meet the requirements of adolescents for “new, fun and meaningful” content in PE [58], and for contributing to the 2030 Agenda for Sustainable Development goals (e.g., good health, quality education, sustainable cities) [59].

In the current study, it was also found that most adolescents spent 2 hours or less in SB during their leisure time. Despite this being in line with the SB data stemming from GSHS studies [10,43] that have used the same or similar samples and methods for measuring SB, this finding contradicts the evidence that SB is highly prevalent among adolescents [3,9,60], even after school [8]. Contrary to several studies, where higher participation in PE has been found to be associated with lower SB [34,36,37], our study revealed that adolescents with higher participation in PE classes were more likely not to meet SB recommendations. Other studies found no associations [39,40]. In addition to the methodological circumstances identified above (i.e., SB specific measures, prevalence differences, moderators), the “activitystat” theory [39,41] might be a plausible explanation for this contradictory finding. This theory suggests an energy expenditure threshold for adolescents, and once reached, the rest of daily time may be compensated with reduced PA and more SB [41]. Studies with robust methodological designs (e.g., RCT, longitudinal) and device-based PA/SB measures (e.g., accelerometry) are needed to fully investigate and improve our understanding of this phenomenon. In addition, exploring adolescents’ perspectives on this topic could be of value to further understand and design meaningful PE strategies for helping adolescents to reduce their SB and increase their PA during leisure time [61]. In this regard, from a practical point of view, teachers can inform adolescents about the SB definition and types (e.g., study vs. recreation screen time), health consequences, and implement practical strategies to reduce SB [62]. In doing so, teachers can take advantage of using digital technologies among adolescents in PE and outside of PE and school (e.g., apps and wearable devices as monitoring tools) [63,64,65].

As in previous research [2,3,12,13], most adolescents in this study did not meet the recommendations for vegetable (76.0%) and fruit (63.5%) consumption. Establishing healthy dietary habits would, therefore, seem to be another priority from a public health perspective. Schools, and particularly PE, might play an important role, especially given that we found that the boys and girls who reported to participate in PE on ≥3 days/week in PE were more likely to meet the vegetable recommendations. Furthermore, compared with those who did not participate in PE, adolescents who attended PE classes also had higher odds of meeting the fruit recommendations. These relationships have not often been explored in the literature but are consistent with the findings of Tassitano et al. [34]. In this regard, again, we advocate the importance of adopting multicomponent approaches and cross-sector collaborations, where school, PE, communities, and families work together to create healthy environments and support the development of positive dietary habits and healthy lifestyles in young people [66,67,68,69].

Overall, 9.5% and 14.6% of adolescents, respectively, had smoked cigarettes and consumed alcohol in the last 30 days. These concerning figures align with other evidence [2,3,11,13,46] and highlight the need to address these behaviours. In previous research, to our knowledge, the specific association between PE participation, smoking, and alcohol consumption has rarely been explored in low-middle-income countries and using national student representative samples. However, in one study conducted in Brazil, no association between participation in PE classes and smoking and alcohol consumption was observed [34]. We found these same results for boys but not for girls. On the one hand, those girls who took PE classes 1–2 days/week were more likely not to smoke, but on the other hand, those girls who attended PE classes on 3 or more days were more likely to smoke and drink alcohol. Considering the limited literature on this topic, the cross-sectional study design of our study, and that these complex behaviours depend on individual, sociocultural, and environmental factors, these results are difficult to explain. Therefore, more research is needed to understand better the relationship and mechanisms between PE, PA, alcohol use, and smoking among specific groups of adolescents from diverse sociocultural and environmental contexts.

Globally, most adolescents live an unhealthy lifestyle and exhibit multiple modifiable risk factors for their current and future health [1,2,4,13]. In the current study, only one in four adolescents adopted ≥5 healthy behaviours. Thus, worldwide, prevention strategies targeting multiple lifestyle health-related behaviours among adolescent students seem to be a priority.

Schools and PE are key contexts for the promotion of healthy lifestyles, and an increased interest in health-focused curriculum activities and interventions in PE was identified [17,18,22,70]. It is noteworthy that, generally, across all regions, participating in three or more PE classes per week was positively associated with adopting ≥5 healthy behaviours. This trend was also observed when all incomes were considered, particularly for the low-, lower-middle-, and upper-middle-income countries. The only exception was for boys of high-income countries, where no associations were identified. Considering world region specificities, independent of the number of PE classes per week and gender, the priority regions to promote healthy behaviours are Africa, the Americas, and the Eastern Mediterranean since those regions presented the lowest odds of adopting ≥5 healthy behaviours. These findings may have important implications for education and health policy. Overall, at a time when curriculum PE time has itself been threatened in many countries [23,32,71], and considering the positive associations found between PE and the adoption of several health-related behaviours [28,30], this study provides further evidence of the potential importance of providing all adolescents with three or more PE lessons per week. These results are therefore also supportive of daily PE recommendations [23,25].

In addition to increasing PE time and the number of PE classes, we reinforce the need to ensure that students are afforded high-quality PE experiences [23,28,30,49]. This study did not consider any indicator of PE quality since this was not available, nor was it the purpose of the GSHS. Further studies should, therefore, consider PE quality indicators [49] and the relationship between the quality of PE students’ experiences and their health-related behaviours. The promotion of health through PE has been discussed [17,18,49,70] and Armour and Harris consider that “the development of new, complex, evidence-based and personalized ‘PE-for-health’ pedagogies is the next major step to be taken in PE research” [70] (p. 201) and practice. We agree that this could help to strengthen the role of PE in promoting healthier lifestyles among adolescents. For this to happen, PE teachers must be better prepared with the specific knowledge, skills, and attitudes to develop and sustain high-quality PE programs, emphasizing diverse health-related PE outcomes that effectively promote healthier lifestyles. In this regard, continuous professional development, innovative opportunities, and the adequate preparation of future PE teachers are key to making progress in this area [17,22,70,72,73].

Another promising approach for promoting healthy lifestyles in PE, as well in other sectors (e.g., sport, public health), that has gained prominence in the last few years is the focus on health and physical literacy [23,52,74]. Indeed, physical literacy has been considered the foundation of PE and healthy lifestyles [23]. Until now, many studies have focused their attention on defining [52,75] and measuring [76,77] physical literacy. Further research efforts are required to better elucidate effective methods of understanding, enhancing and monitoring physical literacy.

This study is not without limitations. The cross-sectional design of the study did not allow causality assumptions to be made between PE classes and health-related outcomes. Association does not mean causality and residual confounding by unmeasured factors is a possibility in any study. Future studies that use a longitudinal and/or a randomized controlled trials design are recommended to provide an improved understanding of the role of PE quantity (and quality) on health-related behaviours among adolescents.

The PE and health-related behaviours were self-reported and, therefore, were subject to biases. The questions used were single-item measures, which can be quite limited when it comes to an in-depth understanding of specific and complex behaviours.

The GSHS draws questions from a self-report questionnaire of the CDC’s Youth Risk Behaviour Survey (YRBS) [47,78,79] applied in the United States. Data on the validity of all self-reported behaviours that are included in the YRBSS questionnaire is limited [78,79]. However, the CDC has conducted two test–retest reliability studies of the national YRBS questionnaire, finding a substantial-to-high reliability in most questions and changing/eliminating those questions with limited reliability [80,81]. Despite the GSHS’s rigorous standardized methodology for adapting, translating, and implementing the questionnaire in each country [42], to our knowledge no available information exists regarding the validity and reliability of the instrument/questions in each country, except for Fijian’s girls [82]. In this study, the average agreement between test and retest was 77%. Thus, research concerning the validity and the reliability of the GSHS questions in diverse contexts and populations is recommended, namely, against device-based measures when applicable and possible (e.g., accelerometry for PA and SB).

Despite the limitations identified above, because of the large samples used in the study in each country, self-reporting was the most feasible methodology to be used for such an epidemiological study [5,83,84]. This approach is often the only option available, namely, in low-to-middle-income countries [5]. Indeed, some of the used questions (e.g., PA, SB, alcohol) were tested for reliability and validity in different settings against device-based measures [84,85,86,87]. Asking about these behaviours was done routinely in epidemiological studies [1,2,4,13,83] and there are numerous published papers using these measures in the context of the GSHS and others [5,12,13,32,46].

Some important PE-related variables to be considered in future research might consider the duration of each PE lesson, teacher’s behaviours, student’s moderate-to-vigorous PA levels in classes, learning tasks, and school characteristics. New research should also use a more comprehensive list of influencing factors for each health-related behaviour (e.g., parental intake and education; family and friend’s influences; body mass index; psychological factors; school, neighbourhood, community, and political level factors). Regarding the health-related behaviours, other indicators could be collected (e.g., PA—intensity; active transport—type of behaviour, distance from home to school, safety, and environmental features; SB type and contexts—screen-related, during school, and leisure; nutrition—portions per day and types of nutrients; alcohol and tobacco—exposure per day, types, and context for consumption). Future studies could also consider other important health-related lifestyle behaviours, such as sleep [9].

The GSHS involves adolescents who are in school. Therefore, health-related behaviour estimates are not representative of the entire adolescent population of a country. Involving out-of-school adolescents is important in future research initiatives. The GSHS data used in this study were collected in different years and each region included a different number of countries. Direct comparisons between countries and world regions should be made with caution. Region estimates might also not be representative of a region since only some countries from each region participated in the GSHS.

This study has several strengths, including the large samples and the external validity of the findings, as well as the focus on samples from diverse income-economy countries and world regions. At the methodological level, relying on standard procures and using the same questions in each country is a positive feature that makes the data comparable. The approach to exploring the relationship between participation in PE classes and several health-related behaviours (rather than just one or two behaviours, as is typical in other literature) is another important strength for advancing the existing evidence base. The evidence stemming from this study is considered of relevance and importance in informing public education and health policies and in helping to identify other pathways and means through which PE can promote healthier lifestyles among young people.

## 5. Conclusions

Globally, few adolescents are living a healthy lifestyle. The findings suggest a positive association between participation in PE classes and a range of health-related behaviours among adolescents. Worldwide, quality PE delivered daily or at least 3 days/week may be associated with and have a positive influence on other health-related behaviours and should be encouraged to promote healthier lifestyles among adolescents.

## Figures and Tables

**Table 1:** Participants’ characteristics.

		Weighted Sample	
	n	$\hat{N}$	% (95% CI)
**Sex**			
Boys	104,207	31,720,517	51.0 (49.7, 52.3)
Girls	117,914	30,482,324	49.0 (47.7, 50.3)
**Age**			
13 years	44,139	13,668,517	22.0 (20.6, 23.4)
14 years	56,226	17,088,216	27.5 (26.1, 28.9)
15 years	54,809	14,639,544	23.5 (22.5, 24.6)
16 years	43,337	10,211,684	16.4 (15.1, 17.8)
17 years	23,610	6,594,880	10.6 (9.3, 12.0)
**Food insecurity (SES proxy report)**			
Never/rarely	58,918	19,146,235	31.1 (30.1, 32.2)
Sometimes/most of the time/always	153,575	42,339,970	68.9 (67.8, 69.9)
**Region**			
African Region	27,788	8,195,278	13.2 (11.9, 14.6)
Americas Region	68,753	6,678,578	10.7 (9.8, 11.8)
Southeast Asia Region	32,695	20,423,844	32.8 (29.8, 36.0)
Eastern Mediterranean Region	32,274	10,447,384	16.8 (14.8, 18.9)
Western Pacific Region	60,611	16,457,756	26.5 (24.2, 28.9)
**Income**			
Low income	27,505	12,274,641	19.8 (17.3, 22.7)
Lower-middle income	75,725	37,824,314	60.7 (58.3, 63.1)
Upper-middle income	89,284	11,452,473	18.6 (17.5, 19.7)
High income	27,569	649,337	0.9 (0.8, 1.0)
**Participation in physical education classes**			
0 days	46,597	11,325,217	18.2 (17.2, 19.3)
1–2 days	118,262	35,244,897	56.7 (54.9, 58.4)
≥3 days	57,262	15,632,727	25.1 (24.0, 26.3)
Physical activity			
Did not meet recommendations	185,772	52,286,483	84.8 (84.0, 85.7)
Met recommendations	34,201	9,342,697	15.2 (14.3, 16.0)
**Sedentary behaviour (sitting time)**			
Did not meet recommendations	84,581	19,397,870	31.6 (30.3, 32.9)
Met recommendations	134,909	42,055,541	68.4 (67.1, 69.7)
**Active travel to school**			
No	102,416	25,472,511	41.3 (39.7, 42.8)
Yes	118,000	36,278,856	58.7 (57.2, 60.3)
**Fruit consumption**			
Did not meet recommendations	138,914	39,022,734	63.5 (62.5, 64.5)
Met recommendations	79,150	22,451,120	36.5 (35.5, 37.5)
**Vegetable consumption**			
Did not meet recommendations	168,865	46,678,937	76.0 (75.1, 76.8)
Met recommendations	49,113	14,760,158	24.0 (23.2, 24.9)
**Smoking cigarettes**			
Yes	25,870	5,554,059	9.5 (8.9, 10.2)
No	176,870	52,708,387	90.5 (89.8, 91.1)
**Alcohol consumption**			
Yes	41,261	7,280,342	14.6 (13.7, 15.5)
No	137,959	42,584,054	85.4 (84.5, 86.3)
**Healthy lifestyle behaviours index**			
0	1156	186,172	0.4 (0.4, 0.5)
1	7082	1,091,078	2.4 (2.2, 2.6)
2	22,975	4,926,778	11.0 (10.3, 11.7)
3	45,624	12,575,587	28.0 (27.2, 28.8)
4	45,763	14,112,531	31.4 (30.7, 32.1)
5	25,205	8,367,151	18.6 (17.9, 19.4)
6	8609	3,087,242	6.9 (6.3, 7.4)
7	1452	577,887	1.3 (1.0, 1.6)

**Table 2:** Relationship between healthy behaviours and participation in PE classes.

	Participation in PE Classes					
	Boys			Girls		
	0 Days/Week	1–2 Days/Week	≥3 Days/Week	0 Days/Week	1–2 Days/Week	≥3 Days/Week
	% (95% CI)	% (95% CI)	% (95% CI)	% (95% CI)	% (95% CI)	% (95% CI)
**Physical activity**						
Did not meet recommendations	88.7 (87.2, 90.2)	82.8 (81.5, 84.0)	73.8 (71.8, 75.8)	92.2 (91.3, 93.1)	89.2 (88.2, 90.1)	83.5 (81.9, 85.0)
Met recommendations	11.3 (9.8, 12.8)	17.2 (16.0, 18.5)	26.2 (24.2, 28.2)	7.8 (6.9, 8.7)	10.8 (9.9, 11.8)	16.5 (15.0, 18.1)
Sedentary behaviour (sitting time)						
Did not meet recommendations	25.2 (23.2, 27.2)	33.6 (31.9, 35.3)	30.0 (28.0, 31.9)	25.3 (23.4, 27.2)	35.0 (33.1, 36.8)	30.1 (24.8, 31.8)
Met recommendations	74.8 (72.8, 76.8)	66.4 (64.7, 68.1)	70.0 (68.1, 72.0)	74.7 (72.8, 76.6)	65.0 (63.2, 66.9)	69.9 (68.2, 71.6)
Active travel to school						
No	52.5 (50.3, 54.6)	37.9 (35.8, 40.1)	34.7 (32.8, 36.7)	54.1 (51.3, 56.9)	40.5 (38.1, 42.9)	40.4 (38.6, 42.2)
Yes	47.5 (45.4, 49.7)	62.1 (59.9, 64.2)	65.3 (63.3, 67.2)	45.9 (43.1, 48.7)	59.5 (57.1, 61.9)	59.6 (57.8, 61.4)
Fruit consumption						
Did not meet recommendations	67.8 (65.3, 70.1)	63.2 (61.5, 64.8)	64.5 (62.6, 66.4)	65.6 (63.5, 67.7)	62.0 (60.6, 63.4)	61.5 (59.9, 63.2)
Met recommendations	32.2 (29.9, 34.7)	36.8 (35.2, 38.5)	35.5 (33.6, 37.4)	34.4 (32.3, 36.5)	38.0 (36.6, 39.4)	38.5 (36.8, 40.1)
Vegetable consumption						
Did not meet recommendations	76.9 (75.0, 78.7)	77.6 (76.3, 78.8)	71.1 (69.4, 72.8)	78.5 (76.8, 80.1)	77.2 (76.0, 78.3)	72.1 (70.6, 73.4)
Met recommendations	23.1 (21.3, 25.0)	22.4 (21.2, 23.7)	28.9 (27.2, 30.6)	21.5 (19.9, 23.2)	22.8 (21.7, 24.0)	27.9 (26.6, 29.4)
Cigarettes						
Yes	14.4 (12.8, 16.1)	14.4 (13.2, 15.7)	14.1 (12.1, 16.4)	4.4 (3.8, 5.2)	4.1 (3.6, 4.7)	6.0 (5.3, 6.8)
No	85.6 (83.9, 87.2)	85.6 (84.3, 86.8)	85.9 (83.6, 87.9)	95.6 (94.8, 96.2)	95.9 (95.3, 96.4)	94.0 (93.2, 94.7)
Alcohol consumption						
Yes	15.5 (14.0, 17.1)	18.6 (17.2, 20.2)	14.6 (13.1, 16.2)	10.9 (9.7, 12.3)	12.5 (11.5, 13.5)	12.0 (10.8, 13.3)
No	84.5 (82.9, 86.0)	81.4 (79.8, 82.8)	85.4 (83.8, 86.9)	89.1 (87.7, 90.3)	87.5 (86.5, 88.5)	88.0 (86.7, 89.2)

**Table 3:** Relationship between meeting healthy behaviour recommendations and participation in PE classes.

	Participation in PE Classes					
	Boys			Girls		
	0 Days/Week OR (95% CI)	1–2 Days/Week OR (95% CI)	≥3 Days/Week OR (95% CI)	0 Days/Week OR (95% CI)	1–2 Days/Week OR (95% CI)	≥3 Days/Week OR (95% CI)
Physical activity Met recommendations	1.00 (ref.)	**1.76** **(1.47, 2.12)**	**2.75** **(2.32, 3.26)**	1.00 (ref.)	**1.67** **(1.41, 1.97)**	**2.34** **(2.01, 2.72)**
Sedentary behaviour (sitting time) Met recommendations	1.00 (ref.)	**0.73** **(0.65, 0.82)**	**0.74** **(0.65, 0.84)**	1.00 (ref.)	**0.73** **(0.66, 0.82)**	**0.76** **(0.68, 0.85)**
Active travel to school Yes	1.00 (ref.)	**1.85** **(1.66, 2.07)**	**2.05** **(1.83, 2.29)**	1.00 (ref.)	**1.74** **(1.52, 1.99)**	**1.73** **(1.53, 1.96)**
Fruit consumption Met recommendations	1.00 (ref.)	**1.18** **(1.05, 1.33)**	**1.19** **(1.07, 1.33)**	1.00 (ref.)	**1.12** **(1.02, 1.24)**	**1.19** **(1.08, 1.32)**
Vegetable consumption Met recommendations	1.00 (ref.)	0.96 (0.85, 1.07)	1.36 (1.21, 1.54)	1.00 (ref.)	1.10 (0.98, 1.23)	**1.42** **(1.26, 1.60)**
Cigarettes No	1.00 (ref.)	**1.06** **(0.92, 1.23)**	0.98 (0.81, 1.18)	1.00 (ref.)	**1.26** **(1.09, 1.47)**	**0.71** **(0.57, 0.88)**
Alcohol consumption No	1.00 (ref.)	0.97(0.85, 1.10)	1.00 (0.89, 1.12)	1.00 (ref.)	1.07 (0.92, 1.25)	**0.84** **(0.73, 0.97)**

Analyses were adjusted by age, socioeconomic status (huger status), region, and country income. Note: Those OR (95% CI) highlighted in bold were significant.

**Table 4:** Relationship between ≥5 healthy behaviours and participation in PE classes, stratified by regions.

Region	≥5 Healthy Behaviours OR (95% CI)					
	Boys			Girls		
	Participation in Physical Education Classes			Participation in Physical Education Classes		
	0 Days/Week	1–2 Days/Week	≥3 Days/Week	0 Days/Week	1–2 Days/Week	≥3 Days/Week
All regions	1.00 (ref.)	**1.43** **(1.24, 1.64)**	**2.09** **(1.77, 2.47)**	1.00 (ref.)	**1.45** **(1.29, 1.63)**	**1.79** **(1.58, 2.03)**
African Region	1.00 (ref.)	**1.24** **(1.01, 1.53)**	**2.01** **(1.62, 2.49)**	1.00 (ref.)	**1.27** **(1.05, 1.54)**	**1.83** **(1.50, 2.24)**
Americas Region	1.00 (ref.)	**1.29** **(1.03, 1.60)**	**1.68** **(1.35, 2.07)**	1.00 (ref.)	1.13 (0.94, 1.36)	**1.50** **(1.19, 1.89)**
Southeast Asia Region	1.00 (ref.)	**1.60** **(1.21, 2.11)**	**2.56** **(1.87, 3.52)**	1.00 (ref.)	**1.60** **(1.29, 1.98)**	**2.09** **(1.61, 2.70)**
Eastern Mediterranean Region	1.00 (ref.)	**1.16** **(0.72, 1.88)**	**1.76** **(1.09, 2.84)**	1.00 (ref.)	1.28 (0.90, 1.82)	**1.80** **(1.06, 3.05)**
Western Pacific Region	1.00 (ref.)	**1.61** **(1.31, 1.99)**	**1.83** **(1.49, 2.24)**	1.00 (ref.)	**2.06** **(1.67, 2.54)**	**2.08** **(1.66, 2.61)**

Analyses were adjusted for age and socioeconomic status (hunger status). Note: Those OR (95% CI) highlighted in bold are significant.

**Table 5:** Relationship between ≥5 healthy behaviours and PE participation, stratified by income (World Bank Classification).

Income	≥5 Healthy Behaviours OR (95% CI)					
	Boys			Girls		
	Physical Education Attendance			Physical Education Attendance		
	0 Days/Week	1–2 Days/Week	≥3 Days/Week	0 Days/Week	1–2 Days/Week	≥3 Days/Week
All incomes	1.00 (ref.)	**1.43** **(1.25, 1.64)**	**2.09** **(1.78, 2.45)**	1.00 (ref.)	**1.45** **(1.29, 1.63)**	**1.79** **(1.58, 2.03)**
Low income	1.00 (ref.)	**1.98** **(1.40, 2.79)**	**2.79** **(2.00, 3.89)**	1.00 (ref.)	**2.02** **(1.63, 2.51)**	**2.24** **(1.74, 2.87)**
Lower-middle income	1.00 (ref.)	**1.30** **(1.12, 1.52)**	**1.52** **(1.31, 1.78)**	1.00 (ref.)	**1.33** **(1.14, 1.56)**	**1.51** **(1.29, 1.78)**
Upper-middle income	1.00 (ref.)	**1.39** **(1.12, 1.74)**	**1.81** **(1.45, 2.26)**	1.00 (ref.)	**1.27** **(1.07, 1.52)**	**1.71** **(1.40, 2.09)**
High income	1.00 (ref.)	1.19 (0.79, 1.57)	1.36 (0.92, 2.01)	1.00 (ref.)	1.18 (0.85, 1.63)	**1.39** **(1.08, 2.06)**

Analyses were adjusted for age and socioeconomic status (hunger status). Note: Those OR (95% CI) highlighted in bold are significant.

## Data Availability

We used publicly available data in this research. At the end of the two-year window, the data set and codebook associated with the core GSHS questionnaire modules were made available to the public on the GSHS website.

## Supplementary Materials

The following supporting information can be downloaded at: https://www.mdpi.com/article/10.3390/ijerph19020955/s1. Table S1: Survey year and sample size for countries that participated in the Global School-Based Health Survey, 2010–2017.
