# Supplementary material for: Participation in Physical Education Classes and Health-Related Behaviours among Adolescents from 67 Countries
Source: Int J Environ Res Public Health. 2022 Jan 15;19(2):955. doi: 10.3390/ijerph19020955 (PMC8775417; doi:10.3390/ijerph19020955)
Supplement: Supplementary file 1 [file ijerph-19-00955-s001.zip › ijerph-1468399-SI.pdf]

Supplementary material

Table S1. Survey year and sample size for countries that participated in the Global School-Based Health Survey, 2010–2017.

Country by Region	Income Classification	Survey Year	Overall Response Rate (%) *	Final Sample **	Weighted % of Girls in Final Sample (95% CI)
**African Region**					
Algeria	Upper-middle	2011	98	3765	49.7 (48.0, 51.3)
Benin	Low	2016	78	1530	31.2 (27.1, 35.5)
Ghana	Lower-middle	2012	76	2142	49.5 (46.2, 52.9)
Liberia	Low	2017	71	1098	48.3 (44.6, 52.0)
Mauritania	Low	2010	70	1810	45.9 (40.6, 51.3)
Mauritius	Upper-middle	2017	84	2887	53.3 (37.4, 68.6)
Mozambique	Low	2015	80	1241	47.5 (43.3, 51.8)
Namibia	Upper-middle	2013	89	3218	55.4 (53.3, 57.5)
Seychelles	Upper-middle	2015	82	1865	51.4 (48.5, 54.4)
Sierra Leone	Low	2017	82	2260	49.6 (43.6, 55.7)
Sri Lanka	Lower-middle	2016	89	3116	51.6 (41.8, 61.3)
Tanzania	Low	2014	87	2856	50.1 (46.9, 53.3)
**Region of Americas**					
Anguilla		2016	88	755	51.4 (46.9, 55.9)
Argentina	Upper-middle	2012	71	25,652	52.5 (50.2, 54.7)
Bahamas	High	2013	78	1058	51.4 (47.4, 55.4)
Barbados	High	2011	73	1548	50.9 (45.8, 56.1)
Belize	Lower-middle	2011	88	1564	51.2 (46.6, 55.7)
Bolivia	Lower-middle	2012	88	3249	49.1 (46.7, 51.5)
Chile	Upper-middle	2013	60	1598	51.7 (45.8, 57.6)
Curaçao	High	2015	83	1884	51.3 (46.8, 55.9)
Dominican Republic	Upper-middle	2016	63	1258	50.1 (45.6, 54.5)
El Salvador	Lower-middle	2013	88	1767	48.2 (43.7, 52.7)
Guatemala	Lower-middle	2015	82	3787	47.2 (43.3, 51.1)
Guyana	Lower-middle	2010	76	2277	51.5 (48.0, 54.2)
Honduras	Lower-middle	2012	79	1440	52.3 (48.3, 56.3)
Jamaica	Upper-middle	2017	60	1553	51.7 (45.7, 57.7)
Lebanon	Upper-middle	2017	82	4255	54.2 (49.2, 59.1)
Paraguay	Upper-middle	2017	87	2713	51.1 (49.0, 53.3)
Peru	Upper-middle	2010	85	2802	49.5 (44.0, 55.1)
Saint Kitts and Nevis	Upper-middle	2011	70	1648	49.5 (49.5, 49.5)
Suriname	Upper-middle	2016	83	1739	50.8 (42.8, 58.7)
Trinidad and Tobago	High	2017	89	2862	52.5 (45.2, 59.7)
Uruguay	Upper-middle	2012	77	3344	54.5 (52.7, 56.3)
**South-East Asia Region**					
Bangladesh	Low	2014	91	2757	33.9 (26.5, 42.2)

Bhutan	Lower-middle	2016	95	5712	53.5 (51.8, 55.2)
Indonesia	Lower-middle	2015	94	8749	50.2 (48.0, 52.3)
Myanmar	Lower-middle	2016	85	2435	53.2 (51.2, 55.2)
Nepal	Low	2015	69	5492	50.7 (48.7, 52.7)
Thailand	Upper-middle	2015	89	4881	52.9 (48.7, 57.0)
Timor-Leste	Lower-middle	2015	79	2669	52.1 (49.8, 54.3)
**Eastern Mediterranean Region**					
Afghanistan	Low	2014	79	1889	46.3 (30.1, 63.3)
Bahrain	High	2016	89	5760	48.3 (34.8, 62.0)
Egypt	Lower-middle	2011	85	2041	50.1 (39.3, 61.0)
Iraq	Lower-middle	2012	88	1811	42.5 (29.6, 56.6)
Kuwait	High	2015	78	3041	49.6 (32.4, 66.8)
Morocco	Lower-middle	2016	91	4758	45.8 (43.1, 48.5)
Qatar	High	2011	87	1349	50.0 (38.2, 61.7)
Sudan	Lower-middle	2012	77	2059	46.8 (30.2, 64.2)
Syria	Lower-middle	2010	97	2551	48.3 (38.7, 58.1)
United Arab Emirates	High	2016	80	5022	51.5 (42.4, 60.5)
Yemen	Lower-middle	2014	75	1993	46.3 (34.7, 58.4)
**Western Pacific Region**					
Brunei Darussalam	High	2014	90	2361	50.1 (45.8, 54.5)
Cambodia	Low	2013	85	2807	49.7 (47.6, 51.8)
Cook Island		2015	65	641	51.1 (46.4, 56.0)
Fiji	Upper-middle	2016	79	2915	50.9 (44.0, 57.7)
Kiribati	Lower-middle	2011	85	1483	53.4 (49.7, 57.0)
Laos	Lower-middle	2015	70	3587	47.0 (44.6, 49.4)
Malaysia	Upper-middle	2012	89	24,923	50% (48.1, 51.9)
Mongolia	Lower-middle	2013	88	4467	51.3 (49.5, 53.2)
Niue		2010	81	102	39.2 (39.2, 39.2)
Philippines	Lower-middle	2015	79	7738	51.2 (47.9, 54.5)
Samoa	Lower-middle	2017	59	1515	54.1 (46.9, 61.0)
Solomon Islands	Low	2011	85	1214	45.5 (41.2, 49.9)
Tonga	Lower-middle	2017	90	2361	49.7 (45.3, 54.1)
Tuvalu	Upper-middle	2013	90	673	51.9 (51.9, 51.9)
Vietnam	Lower-middle	2013	96	3005	53.2 (51.0, 55.3)
Wallis and Futuna	High	2015	82	819	53.6 (48.0, 59.1)

** Students aged 13–17 years with complete data on PE, sex, age and with data on 'weighting' the analysis; * from countries with more than 99 participants.
